# Blood Biochemical Acute Daily Responses During an Intensive Training Microcycle: A Case Report of an Olympic Kayaker

**DOI:** 10.3390/sports14080325

**Published:** 2026-08-01

**Authors:** José Augusto Rodrigues dos Santos, Rodrigo Zacca, David Gomes Fernandes, Pedro Silva Oliveira, Diogo Duarte Carvalho

**Affiliations:** 1 Centre of Research, Education, Innovation and Intervention in Sport (CIFI2D), Faculty of Sport (FADEUP), University of Porto, 4200-450 Porto, Portugal; 2 Research Center in Physical Activity, Health and Leisure (CIAFEL), Faculty of Sports, University of Porto (FADEUP), 4200-450 Porto, Portugal; 3 Laboratory for Integrative and Translational Research in Population Health (ITR), 4050-600 Porto, Portugal; 4 Laboratory of Sport Physiology, Faculty of Sport (FADEUP), 4050-600 Porto, Portugal; 5 Nucleus of Research in Human Motricity Sciences, Universidad Adventista de Chile, Chillán 3780000, Chile

**Keywords:** single subject design, sprint-canoe, elite athlete, high performance, athlete monitoring

## Abstract

Monitoring physiological responses to intensified training is critical in elite sport, yet evidence in world-class kayak paddlers remains limited. To assess biochemical and hematological acute responses during an intensified training microcycle in an elite flat-water kayaker, a 35-year-old Olympic-level kayaker was monitored across two consecutive 7-day microcycles (pre-intensified and intensified). During the intensified microcycle, fasting venous blood samples were collected over five consecutive mornings. Hematological, biochemical, and hormonal markers were analyzed using standardized laboratory procedures. Training intensification induced marked but physiological changes. From Monday to Tuesday, leucocytes increased from 5.7 to 7.3 × 10^9^/L (+28%), while creatine kinase (CK) rose from 291 to 602 U/L (+107%) and remained elevated (579–604 U/L). Serum iron decreased from 131 to 88 µg/dL (−32.8%) and remained below baseline throughout the microcycle. Triglycerides declined from 63 to 52 mg/dL (−17.5%) and stabilized thereafter. AST increased from 33 to 41 U/L (+24.2%) with minor fluctuations. Across the microcycle, cortisol rose from 522 to 575–578 nmol/L (+10–10.7%), whereas testosterone remained stable (29.4–31.2 nmol/L). Epinephrine increased from 13 to 37 ng/L (+184.6%). Erythrocytes, hemoglobin, and C-reactive protein remained stable. The intensified microcycle elicited substantial but controlled physiological perturbations, reflecting increased muscle stress and systemic load without evidence of maladaptation.

## 1. Introduction

Training in high-performance sport is systematically designed to induce physiological and functional adaptations that allow athletes to reach a new functional threshold and tolerate progressively higher loads [1]. This adaptive process is inherently cyclical, with periods of increased training stress and transient performance decline followed by phases of supercompensation and enhanced physical capacity [2]. At the biological level, these changes emerge from complex molecular responses to repeated exercise stimuli, including the activation and repression of signaling pathways that regulate gene expression, protein turnover, and tissue remodeling [3,4].

Skeletal muscle is a highly plastic tissue [5] that continuously adjusts protein synthesis and degradation in response to perturbations of cellular homeostasis induced by training [6]. Variations in training volume [7], intensity [8], and frequency [9], alongside recovery strategies [10] and nutritional status [11], modulate the direction and magnitude of these adaptations. Over time, the interplay between mechanical load [12], metabolic stress [13], and endocrine responses [14] contributes to the development of sport-specific phenotypes that support performance in elite athletes [3,4]. However, when training loads are not appropriately managed, the same stimuli that drive adaptation can also lead to excessive fatigue, maladaptation, or even overreaching and overtraining [15].

For coaches and sport scientists, monitoring training load and its physiological consequences is therefore essential to optimize performance while minimizing the risk of negative outcomes [16]. Alongside external- and internal-load measures, blood-based biomarkers have been proposed as valuable tools to characterize the acute and chronic responses to training [17]. Parameters such as hematologic indices, serum enzymes, lipid profile, inflammatory markers, and stress-related hormones may provide insight into muscle damage, metabolic strain, inflammatory status, and anabolic–catabolic balance in athletes [18]. Several studies have examined biomarker dynamics in endurance and team sports across training camps or competition periods, suggesting that changes in markers like creatine kinase, aspartate aminotransferase, cortisol, and catecholamines can help identify excessive strain or insufficient recovery [19].

Flat-water kayaking is a highly demanding Olympic sport that combines high-intensity endurance bouts on water with substantial strength training on land [20]. Elite kayakers typically perform multiple weekly sessions that integrate heavy resistance exercises and high-intensity paddling intervals, often within tightly scheduled preparatory and pre-competitive periods [21]. This training structure imposes considerable neuromuscular and metabolic demands, particularly through repeated upper-body loading and short recovery windows between sessions. Traditional monitoring strategies that rely on sporadic, pre-and-post block blood sampling frequently fail to capture acute homeostatic shifts, thereby missing crucial windows of transient muscle stress or acute non-functional strain. In this context, daily monitoring may help describe the acute responses during intensified microcycles. Despite this practical relevance, detailed information on the day-to-day biochemical responses to intensive microcycles in world-class kayakers remains scarce [22].

Understanding how a highly trained athlete responds to a short period of intensified training may provide important insights for individualizing load prescription. As athletes progress to higher performance levels, inter-individual variability in biomarker responses becomes more relevant, and generalized reference values may be less informative for guiding practice [23], representing a major limitation in traditional workload monitoring. Indeed, current sports science literature emphasizes that an athlete’s biological response is highly contextualized, influenced by unique training histories, adaptive capacities, and sport-specific homeostatic thresholds, irrespective of the athletic discipline. Consequently, relying solely on generic clinical ranges often lacks the sensitivity required to detect early signs of functional overreaching or subtle maladaptation’s. This highlights a crucial knowledge gap: the need for baseline individualization. Case studies in elite performers can therefore contribute to refining the interpretation of biochemical markers and to developing more tailored monitoring strategies that reflect the specific demands of a given sport, position, and phase of the season [24].

In this context, the present case study aimed to evaluate the daily changes in blood biochemical and hematologic markers during consecutive days of an intensive training microcycle in an elite flat-water kayaker, following a pre-intensified microcycle used to contextualize the preceding training structure and establish baseline values. The focus was placed on indicators related to muscle damage, metabolic status, inflammation, iron metabolism, and hormonal and catecholaminergic responses. By describing the pattern and magnitude of these changes while training loads were deliberately increased, this study seeks to provide practical information for coaches and practitioners on how to interpret biochemical data when planning and controlling intensive microcycles in elite kayaking.

## 2. Materials and Methods

### 2.1. Methodological Design

This study employed an observational, longitudinal, single-case with repeated measures design to characterize acute daily biochemical, hormonal, and hematologic responses during an intensified training microcycle in an Olympic-level flat-water kayaker. This design was selected because repeated physiological monitoring under real-world training conditions is difficult to obtain in world-class athletes and may provide useful individualized information that is not captured by group-based approaches. The aim was descriptive and exploratory, focusing on the temporal pattern of biomarker responses within this specific athlete and training context.

### 2.2. Participant

**Figure 1:**
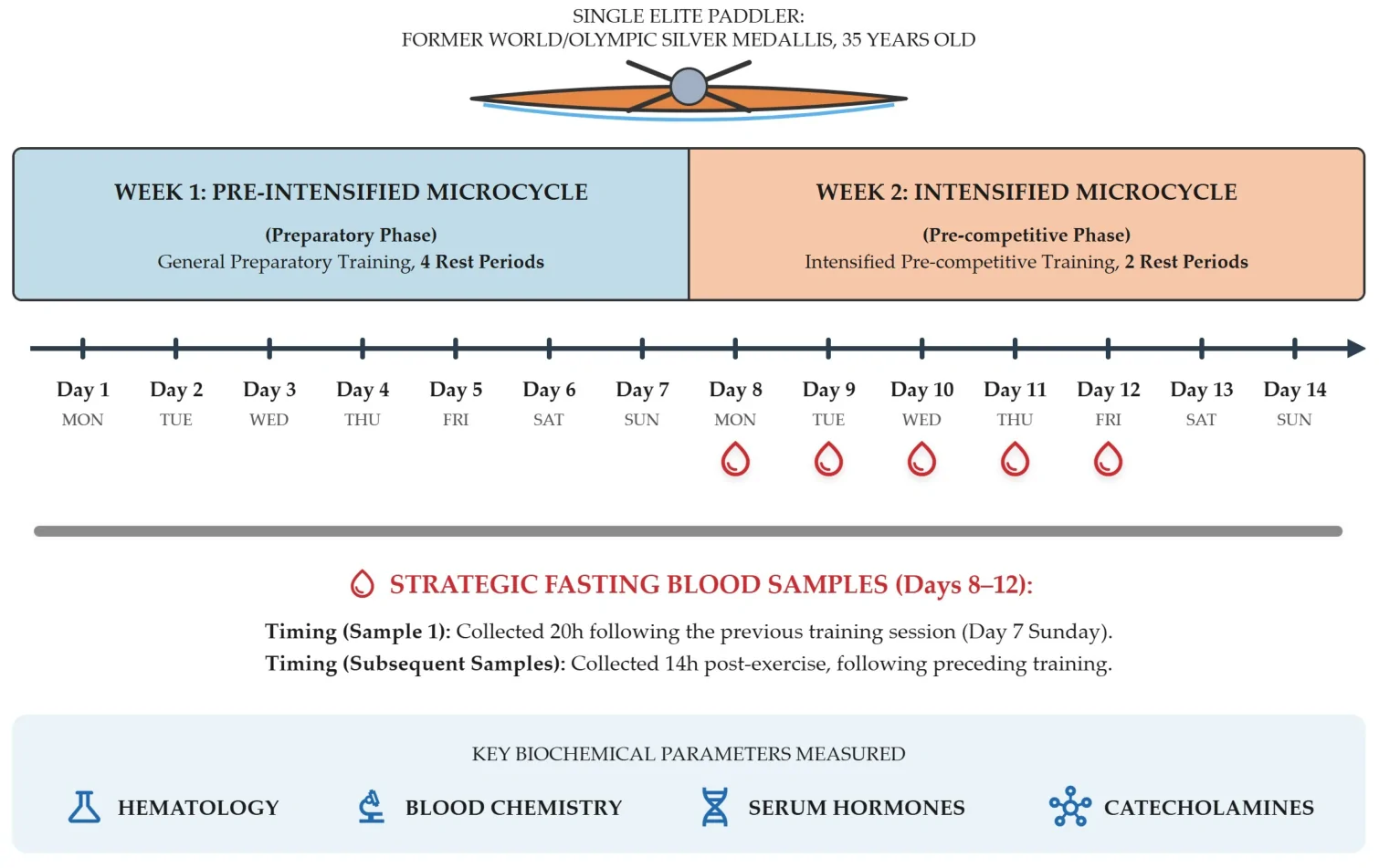
Protocol timeline and venous blood assessments.

A 35-year-old male elite kayak paddler with over 15 years of competitive experience at the highest international level participated in the study. The participant is a former World Champion, European Champion, and Olympic silver medalist in the K2 1000 m event, and at the time of data collection was preparing to qualify for the forthcoming Olympic Games. The kayak paddler was monitored during two 7-day microcycles (one pre-intensified and other intensified weeks) with venous blood collection during the intensified week (see Figure 1). Biometric assessments indicated a height of 185 cm and a stable body mass of 87 kg throughout the monitored training period (two microcycles). Body weight was measured daily under standardized conditions (each morning, in a fasted state, prior to the first training session), with the participant wearing only underwear to ensure longitudinal consistency. To ensure a consistently hydrated state before testing sessions, hydration status was pragmatically monitored on a daily basis via qualitative physical inspection of morning urine color. Dietary intake was retrospectively estimated from the meals and foods reported by the athlete as being consumed during the monitored period. The approximate daily iron intake was calculated from these reported food sources. No formal weighed food record or standardized 24 h dietary recall was performed. The athlete was fully informed of all procedures, risks, and benefits associated with participation, and provided written, informed consent. The study protocol was approved by the institutional ethics committee and all procedures complied with the Declaration of Helsinki and contemporary ethical guidelines for human research [25].

### 2.3. Biochemical Analyses

Blood samples were collected over five consecutive mornings (during the intensified microcycle) under fasting conditions to minimize diurnal and nutritional interference. The baseline sample was obtained on Monday morning, 20 h after the previous training session performed on Sunday (running session of microcycle 1), and was used as the reference sample before the start of the intensified microcycle. All subsequent samples were collected on consecutive mornings, exactly 14 h after the previous training day, under fasting conditions. This sampling schedule was chosen to standardize the overnight recovery period, reduce acute nutritional and diurnal influences, and capture early recovery responses in markers known to remain elevated after exercise, such as CK and AST.

Hematological parameters were analyzed using fluorescence flow cytometry (Sysmex XT-4000i, Sysmex Corp., Kobe, Japan). Blood chemistry analyses were performed by molecular absorption spectrometry using an ADVIA 1800 Chemistry System (Siemens Healthineers, Erlangen, Germany). Serum testosterone and cortisol concentrations were measured by immunochemiluminescence on an ADVIA Centaur XP Immunoassay System (Siemens Healthineers), and catecholamine concentrations were determined using high-performance liquid chromatography (HPLC) to ensure the highest sensitivity for neuroendocrine markers in an elite athletic population. Plasma catecholamines (epinephrine and norepinephrine) were strategically assessed exclusively at baseline (Monday) and at the cumulative completion of the microcycle (Friday). This pre-to-post design was implemented due to the distinct pre-analytical demands, biochemical volatility, and rapid degradation pathways characteristic of catecholaminergic markers, which require immediate specialized refrigerated centrifugation and ultra-low temperature storage (–80 °C).

### 2.4. Training Schedule and Content

The two microcycles corresponded to the final phase of the preparatory period and the initial phase of the pre-competitive period. The criteria used to define the training intensity zones applied during both microcycles are presented in Table 1. The pre-intensified microcycle (Table 2) consisted of ten training sessions, including seven in-water sessions, two strength-training sessions, and two running sessions, interspersed with four rest periods. The subsequent intensified microcycle (Table 3) comprised twelve training sessions, including nine in-water sessions, three strength-training sessions, and one running session, interspersed with two rest periods. Training sets were strictly prescribed according to the corresponding strokes per minute (spm) associated with each intensity zone [26], and kayaking external training load, i.e., volume (km) and intensity (volume × strokes per minute) assessed for the water workouts [26].

### 2.5. Data Analysis

Data were analyzed descriptively due to the single-case design. Absolute values and relative percentage changes were calculated to describe day-to-day fluctuations in hematological, biochemical, hormonal, and catecholaminergic variables during the intensified microcycle. Monday was used as the baseline reference for percentage change calculations, as it represented the sample collected after the pre-intensified microcycle and immediately before the start of the intensified microcycle. Given the sample size (*n* = 1), inferential statistical analyses were not performed. Results were interpreted in relation to the athlete’s within-microcycle pattern, laboratory reference intervals, and previous athlete-monitoring literature. Laboratory reference ranges were used as a clinical safety framework to contextualize the biomarker responses, but not as the sole criterion to define optimal adaptation or recovery. To minimize pre-analytical variability, all blood samples were collected under standardized fasting conditions and at fixed post-exercise intervals.

## 3. Results

**Figure 2:**
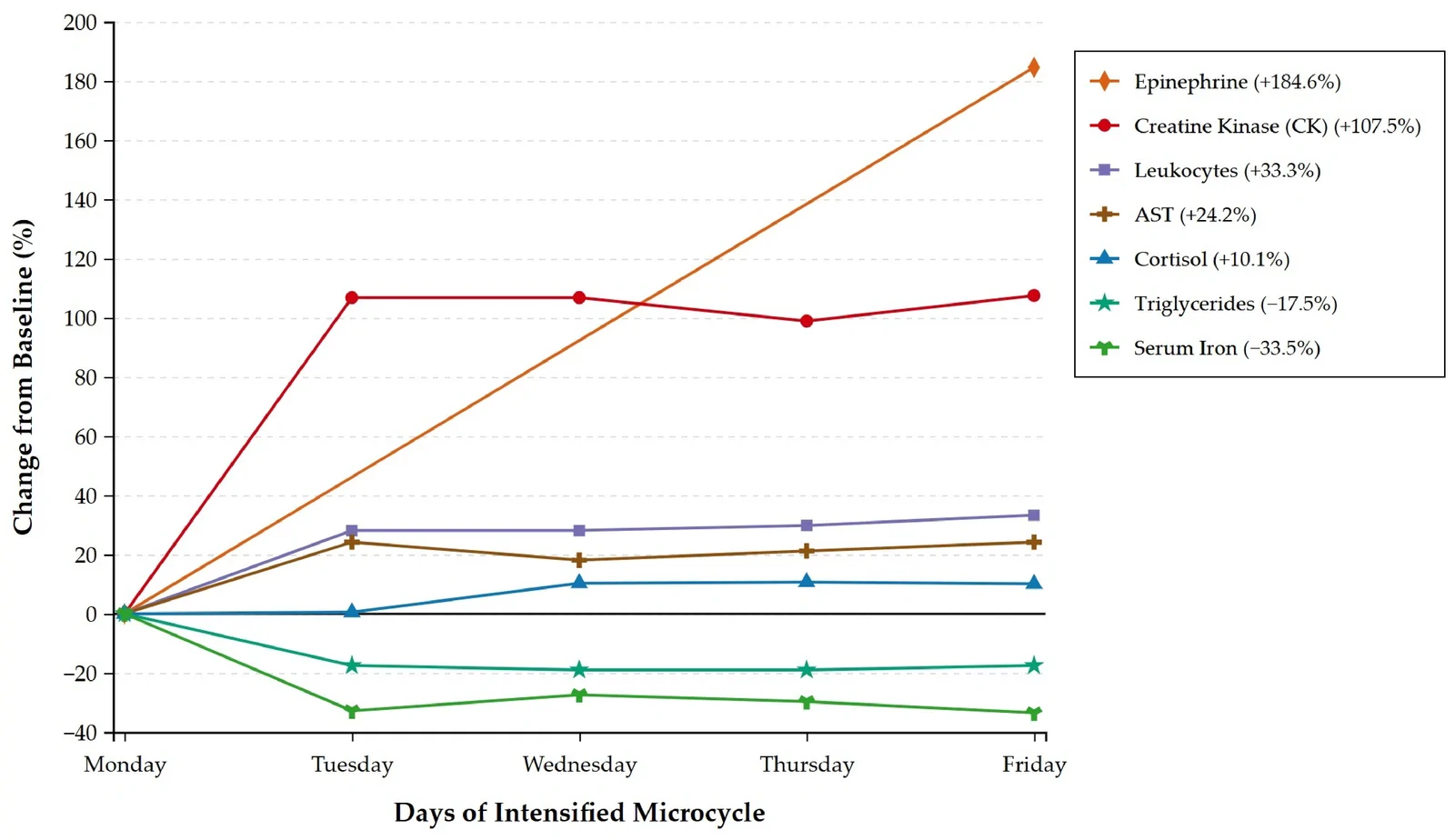
Temporal patterns of selected biomarkers during the intensified training microcycle.

The hematologic and biochemical changes during the intensified microcycle are presented in Table 4 and most clinically relevant variables patterns are shown in the Figure 2. Training intensification led to an increase in leukocyte count, primarily due to a rise in neutrophils, with minor fluctuations observed in eosinophils, basophils, and lymphocytes, while monocytes showed a slight decrease. Serum iron and plasma triglycerides decreased, whereas CK, AST, cortisol and epinephrine levels increased. The remaining biomarkers exhibited notable stability. All changes remained within the physiological range. When expressed as percentage changes, from Monday to Tuesday several variables exhibited substantial alterations. Leucocyte counts increased notably (+28%), while serum iron showed a marked decrease (−32.8%). The largest relative change was observed in creatine kinase (CK) (+107%) while triglycerides decreased moderately (−17%) and AST increased (+24%).

From Tuesday onwards, most variables demonstrated relatively smaller day-to-day fluctuations. Leucocytes remained stable with minimal variation (0–3% changes). Triglycerides remained relatively stable after the initial drop, with negligible daily changes. Serum iron showed a slight rebound on Wednesday (+8%), followed by minor decreases through Friday. CK stayed elevated, with only small oscillations (−4% to +4%). AST fluctuated modestly (−5% to +3%) after its initial rise. In contrast, epinephrine (measured only twice) showed a marked increase on Friday compared to Monday (+185%) but still in low values of the normal range (0–140 ng/L). The remaining biomarkers exhibited relative stability, although potassium showed transient values above the laboratory reference range on Monday (baseline) and Wednesday.

## 4. Discussion

The current case study showed that a five-day intensive training microcycle in an elite flat-water kayaker elicited clear yet physiologically normal fluctuations in several blood biochemical and hematological markers. Daily monitoring revealed increases in indicators of muscle damage and physiological stress, particularly muscle enzymes, stress-related hormones, leukocytes, and catecholamines, while all values remained within established laboratory reference ranges. The reported changes from baseline suggest that the intensified microcycle was associated with acute physiological strain, without evident biochemical, hematological, or clinical signs of maladaptation or pathological fatigue in this elite kayaker [29,30].

The hematological profile provides critical information about the athlete’s response to the microcycle. In the present study, while hematocrit and hemoglobin levels for the flat-water kayaker remained within standard clinical ranges, they were notably higher than values previously reported for elite cyclists during both competitive and non-competitive periods [31]. This discrepancy suggests that the endurance demands of elite kayaking may elicit a distinct hematological response compared to the high-volume training typical of middle- and long-distance runners or road cyclists, which is frequently associated with hemodilution and a subsequent decrease in red blood count indicators [32]. Furthermore, the stable levels of hemoglobin and hematocrit following the microcycle in this study contrast with the findings of Corsetti et al. [33], who observed significant reductions in these markers after 12 days of endurance training. These results likely reflect differences in exercise modality, training duration, or the specific metabolic demands of the upper-body dominated work in kayaking.

Beyond the erythrocytic profile, changes in leukocyte counts are often observed after intensive training and can reflect both transient inflammatory responses and catecholamine-mediated demargination of leukocytes [34]. In the present work, leukocytosis was evident at specific points during the microcycle, coinciding with higher training loads and elevated adrenaline concentrations (epinephrine 13 to 37 ng/L). This pattern is more likely to be compatible with an acute stress response rather than with infection or chronic inflammation, particularly because values remained within reference ranges and no clinical symptoms were reported. Supporting this interpretation, C-reactive protein, an inflammation marker, showed remarkable stability, indicating the kayaker’s ability to adapt to the intensifying training loads [35]. Furthermore, erythrocyte-related parameters showed minor variations which is important for maintaining oxygen transport and performance in endurance-based sports such as kayaking [36].

Although baseline values for various muscle enzymes in the serum show considerable variation across athletes [37], it is crucial to focus on individual variation at different times throughout the season and before and after specific training regimens. In the present study, creatine kinase (CK) levels, measured 20 h after the last pre-competitive training session, rose sharply and remained elevated throughout the microcycle, indicating increased sarcomeric stress or damage [38]. Several studies have shown that CK increases after exertion, depending on individual training levels [39] and exercise type [40], with eccentric contractions producing the highest post-exercise enzyme activity [41]. Aspartate aminotransferase (AST), an enzyme with high muscular expression, exhibited a similar pattern to CK, while alanine aminotransferase (ALT), which is more liver-specific, remained stable at baseline. Monitoring these three enzymes proved to be an effective method for tracking training-induced stress. Although glucose levels remained stable, serum iron dropped 32.8% on the second day and remained below baseline for the rest of the monitoring period, while still falling within normal laboratory reference ranges (80–180 mcg/dL). This reduction may be attributed to intensified training, which increases iron loss through sweat, urine, and the gastrointestinal tract [42], though this is counterbalanced by dietary iron intake. Based on the meals and foods retrospectively reported by the kayaker, dietary iron intake was estimated to be approximately 20 mg/day, being above the Dietary Reference Intake (DRI) of 8 mg [43], justifying the stabilization within normal ranges. However, without ferritin, transferrin saturation, hepcidin, or total iron-binding capacity, the mechanisms underlying this response cannot be fully determined. Therefore, this finding should be interpreted descriptively rather than as evidence of a specific alteration in iron regulation.

In parallel, electrolytes such as sodium and chlorides, remained stable throughout the microcycle, whereas potassium showed transient elevations above the laboratory reference range on Monday and Wednesday. The highest value was observed on Wednesday (5.9 mEq/L), coinciding with sustained CK elevation, which may reflect exercise-induced potassium efflux from contracting skeletal muscle and increased muscle membrane stress during the intensified training period. Importantly, potassium returned to values within the reference range on Thursday and Friday, suggesting a transient physiological response rather than a sustained electrolyte disturbance. These findings are consistent with previous reports in elite athletes, where serum potassium upper limits have been described as higher than those observed in the general population [37].

In this study, the slight decrease in plasma triglycerides and stable cholesterol levels indicate efficient metabolic adaptation to intensified training loads. This consistency aligns with a previous study [44], who observed that even dramatic increases in aerobic volume resulted in triglyceride changes without clinical significance. While regular exercise typically enhances lipid mobilization, the maintenance of these markers within physiological ranges suggests a high homeostatic capacity in trained cohorts [44]. Furthermore, the 24 h rest period prior to blood collection likely allowed for the recovery of baseline values, as observed in similar elite endurance groups.

The endocrine response, specifically the interplay between testosterone (T) and cortisol (C), provides a deeper characterization of the anabolic–catabolic balance during the microcycle [45]. Both hormones are critical regulators of protein and carbohydrate metabolism, acting as competitive agonists at the receptor level within muscle cells [46]. The T/C ratio is widely utilized as a biomarker to gauge physiological strain, typically decreasing in response to increased training intensity and volume, yet remaining reversible through adequate recovery [46]. In the current study, we observed a downward shift in the T/C (see Table 4); however, this change occurred within a very narrow absolute range and falls within the expected cumulative analytical variability associated with the calculation of the ratio. Therefore, it should be interpreted as a minor numerical fluctuation rather than a physiologically meaningful shift, and does not suggest that the training stimulus exceeded the kayaker’s capacity for homeostatic regulation.

The kayaker’s baseline C and T concentrations were comparable to those reported in elite rowers during one preparatory period, with cortisol concentrations ranging from 474 ± 115.1 to 560.7 ± 78.2 nmol/L and testosterone concentrations from 26.0 ± 4.2 to 29.8 ± 8.0 nmol/L between the fourth and twentieth week with increasing training volume [47]. Similarly, values reported for elite soccer players, after conversion to equivalent units (228.9 ± 41.4 ng/mL for C and 13.6 ± 3.3 ng/mL for T), resulted in a comparable T/C ratio of 0.06 ± 0.02 [48]. Together, these similarities in hormonal profiles may reflect the sport-specific training demands and physiological adaptations characteristic of elite athletes.

Although cortisol concentrations rose slightly during the intensified microcycle, it contrasts with the decreases found in well-trained cyclist after a week of intensified training (153 ± 16 to 130 ± 11 ng/mL) although not statistically significant [49]. In our study this elevation should reflects a functional activation of the hypothalamic–pituitary–adrenal axis and the necessary mobilization of energy substrates. Importantly, the observed cortisol fluctuations did not reach thresholds typically associated with maladaptive overreaching or overtraining. The stability of the T/C ratio indicates that the catabolic impact of the intensified microcycle did not severely compromise the anabolic milieu. These findings are broadly consistent with literature on high-performance endurance and team-sport athletes, where moderate, short-term endocrine shifts are expected during intensified blocks but return to baseline given appropriate recovery [50,51].

The analysis of catecholamine responses further elucidates the athlete’s physiological adaptation, supporting the notion that the microcycle successfully increased sympathoadrenal activity without imposing excessive systemic strain. While plasma concentrations of noradrenaline and dopamine typically increase significantly post-exercise before returning to baseline within a few hours [52,53], our findings revealed relatively stable levels of these markers. In contrast, adrenaline concentrations exhibited a notable increase on the final day of the microcycle, aligning with the highest training loads. These elevated adrenaline levels likely reflect the necessary activation of cardiovascular, metabolic, and thermoregulatory systems required to meet the demands of high-intensity exercise [53]. Crucially, as these concentrations remained within established clinical reference ranges, they do not indicate pathological significance or sustained dysregulation of the stress response. When integrated with the biochemical and hematological findings, this pattern suggests an acute sympathoadrenal response to the intensified microcycle, without evidence of sustained dysregulation in the markers assessed, and is compatible with the transient physiological perturbations expected during intensive training in highly trained athletes [54]. These interpretations should be confirmed in future studies comparing biomarker responses during intensified and regular training-load microcycles, and in larger cohorts of elite athletes of both sexes.

The observed pattern of responses are in line with contemporary models describing training adaptation as a sinusoidal process, where periods of higher stress and transient functional decline are followed by supercompensation and enhanced performance capacity [4]. In the present microcycle, the combination of heavy resistance training and high-intensity paddling sessions likely imposed substantial mechanical and metabolic strain on skeletal muscle. The maintenance of enzyme concentrations within conventional laboratory ranges provides clinical context for the observed response, but should not be interpreted as direct evidence of adequate adaptation or training-load tolerance. In elite athletes, “normal” values may not fully capture the functional relevance of biochemical changes, as individualized baselines and sport-specific patterns can differ from those of the general population [17,55]. Nonetheless, the absence of extreme elevations in muscle damage markers, together with stable clinical status and completion of the prescribed training sessions, supports the interpretation that the microcycle was completed without evident biochemical, hematological, or clinical signs of maladaptation in this athlete.

When interpreting biomarker fluctuations for monitoring, non-training sources of variability must also be considered. Nutritional status, circadian rhythm, hydration status, recent exercise, and analytical variability may all influence circulating biomarker concentrations. In the present study, blood collection was standardized through morning fasting sampling and fixed post-exercise collection windows to reduce some of these sources of variation. Nevertheless, these procedures do not eliminate biological or analytical variability. Therefore, biomarker changes should be interpreted cautiously and in combination with the kayaker’s baseline profile, the direction and magnitude of changes, and the training context. The present interpretations considered not only whether values were within or outside laboratory limits, but also the kayaker’s baseline values, the direction and magnitude of changes, the transient or sustained nature of the responses, and evidence from previous athlete-monitoring studies.

### 4.1. Limitations

Despite its strengths, this study has several limitations that must be acknowledged. First, the single-case design precludes generalization to all elite kayakers or to athletes from other sports. Because the analysis was limited to descriptive values and relative percentage changes, the findings do not provide an inferential statistical basis for broader conclusions. The responses observed here are specific to one world-class paddler, within one particular phase of the season, and under a specific microcycle design. Nevertheless, the study provides rare ecological data that may help contextualize individualized biomarker monitoring in high-performance sport. Second, only a selected panel of biochemical and hematological markers was assessed, where additional variables such as cytokines, markers of oxidative stress, or more detailed hormonal profiles might provide further insight into the mechanisms underlying adaptation to intensive training. Third, performance outcomes were not systematically evaluated, which limits the ability to directly relate biochemical changes to functional performance gains, maintenance, or impairment. Additionally, although the prescribed stroke-rate zones were linked to physiological intensity domains based on %HRmax, %VO_2_max, and blood lactate, objective session-by-session internal-load measures such as heart rate, session-RPE, or TRIMP were not collected. Thus, the study characterizes the external intensity structure of the microcycle but cannot directly quantify the athlete’s internal load during each session.

### 4.2. Future Directions

Future research should extend this approach to larger samples of elite kayakers and other endurance or mixed-modal sports, combining repeated biochemical assessments with detailed external- and internal-load monitoring and standardized performance tests. Longitudinal studies across entire macrocycles would help clarify how short-term intensive microcycles contribute to season-long performance trajectories and how individual biomarker profiles can guide personalized load management. Additionally, integrating biochemical data with subjective measures, wearable technology outputs and performance indices may support the development of practical decision-support tools for coaches working in high-performance environments. Such approaches may also help establish biomarker-specific smallest worthwhile change (SWC), minimal clinically important change (MCIC), or minimal detectable change (MDC) thresholds for elite kayaking and similar high-performance contexts.

## 5. Conclusions

This case study shows that a five-day intensive training microcycle can induce substantial but physiologically normal fluctuations in blood biochemical and hematological markers in a world-class flat-water kayaker. Increases in muscle damage enzymes, metabolic and stress-related markers, leukocytes, and catecholamines reflected the acute strain of the microcycle but remained within reference ranges and were not accompanied by clinical signs of maladaptation. Although limited to a single case, these findings suggest that individualized biomarker profiling may help monitor the physiological effects of intensive microcycles in Olympic-level kayaking contexts. However, biomarker monitoring should not be interpreted in isolation and should be integrated with internal-load measures, performance indicators, recovery markers, and athlete-reported outcomes before being used to inform training decisions.

## Figures and Tables

**Table 1:** Definition of training zones based on strokes per minute (spm), percentage of maximal heart rate (%HRmax), percentage of maximal oxygen uptake (%VO_2_max), and blood lactate concentration (adapted from [27,28]).

Training Intensity Zones		SPM	% HRMAX	%VO_2max_	Blood Lactate
**ZONE 1**	**T0**	<58	≤65	≤65	≤1
	**T1**	62–70	70	65	≥1
**ZONE 2**	**T2**	72–80	75	75	≤2
			80	75	≥2
**ZONE 3**	**T3**	82–90	90	85	≤4
			95	90	≥4
**ZONE 4**	**T3+**	98–106	100	100	≤6
ZONE 5 *	**T4**	114–122	-	-	≥6
	**T5**	128–136	-	-	≤8
	**T6**	≥140	-	-	>8

* May not reach maximal heart rate. At these intensities, although efforts are maximal or near-maximal, set durations are shorter and recovery periods are longer.

**Table 2:** Training distribution over the pre-intensified microcycle.

Day	Morning	Afternoon	Total Water Volume (km)	Total Water Intensity (km × SPM)
Monday	Rest	Rest	-	-
Tuesday	Water. 16 km. Aerobic endurance (70/75 spm).	Water. 10 km (Aerobic endurance). (65 spm). Running 30′.	26	1870
Wednesday	Water. 15 km. 7 × 1000 m (500 m at 80/85 spm; 500 m at 85/90 spm), rest 3′.	Gym (Strength). 8 × 20 reps/rest 1′, 55% Maximum Load + Abs/Lumbars.	15	1133
Thursday	Water. 12 km (60/65 spm). 6 × 10″/rest 1′50″. Maximum pace. Start stopped.	Rest.	13	980
Friday	Water. 15 km. 8 × 1′ (95 spm) rest 1′. 6′ recovery. 8 × 45″ (100 spm) rest 1′15″. 3 × 6′ (75 spm) rest 2′.	Gym (Strength). 6 exercises × 8 RM × 7 sets + Abdominals/Lumbars.	18	1250
Saturday	Water. 15 km. 4 × 4′ (2′ at 80 spm; 2′ at 85 spm), res 4′.	Water. 8 km. Easy pace (65 spm).	23	1595
Sunday	Running 1 h.	Rest.		

Abbreviation: spm (strokes per minute). All training sessions end with 15′ of passive stretching.

**Table 3:** Training distribution over the intensified microcycle.

Day	Morning	Afternoon	Total Water Volume (km)	Total Water Intensity (km × SPM)
Monday	Water. 15 km. 6 × 250 m (115/120 spm), rest 5′.	Gym (Strength). 6 exercises × 6 RM × 6 sets + Abdominals/Lumbars.	15	1155
Tuesday	Water. 15 km. 2 × 1000 m/rest 8′ (250 m at 85 spm, 500 m at 90 spm, 250 m at 95 spm) + 2 × 750 m/rest 8′ (250 m at 115 spm, 250 at 110 spm, 250 m at 115 spm).	Water. 10 km. Easy pace. 65 spm.	28.5	1988
Wednesday	Water. 15 km. 8 × 45″ (110 spm) 1′15″ rest. Recovery 6′. 8 × 30″ (115 spm), 1′30″ rest.	Gym (Strength). 6 × 20 reps/rest 40″, 55% Maximum Load + Abs/Lumb. 30′ running.	15	1445
Thursday	Water. 15 km. 6 × 10″/rest 1′50″. Maximum pace. Start stopped.	Rest.	16	1350
Friday	Water. 15 km. 2 × 1000 m/rest 8′ (250 m at 85 spm, 500 m at 90 spm, 250 m at 95 spm) + 2 × 750 m/rest 8′ (250 m at 115/120 spm, 250 at 110/115 spm, 250 m at 115/120 spm).	Gym (Strength). 6 exercises × 6 RM × 5 sets + Abdominals/Lumbars.	15	1338
Saturday	Water. 15 km. 7 × 50″ at 105 spm/1′10″ rest. Recovery 6′ + 7 × 35″ at 110 spm/1′25″ rest.	Water. 8 km. Easy pace (65 spm).	23	1155
Sunday	Water. 10 km. Easy pace (65 spm).	Rest.	10	650

Abbreviation: spm (strokes per minute). All training sessions end with 15′ of passive stretching.

**Table 4:** Hematologic and biochemical changes during the intensified microcycle.

Indicators	Reference Values	Monday (Baseline)	Tuesday	Wednesday	Thursday	Friday
Erythrocytes (10^12^/L)	4.4–6.0	5.2	5.2	5.4	5.3	5.4
Hemoglobin (g/dL)	13.0–18.0	16.4	16	16	16.1	16
Hematocrit (%)	43–55	47.9	46	47.2	46.8	47
MCV (fL)	87–103	88.2	87.8	88.2	87.9	88.1
MCH (pg)	27–35	30.2	30.5	29.9	30.2	30
MCHC (g/dL)	28–36	34.2	34.8	33.9	34.1	34.2
Leucocytes (10^9^/L)	4.0–11.0	5.7	7.3	7.3	7.4	7.6
Neutrophils (%)	53.8–69.8	56.7	60.3	59.4	61	60.8
Eosinophils (%)	0.6–4.6	2.6	1.6	2.1	2.2	2.1
Basophils (%)	0.0–1.5	0.2	0.5	0.6	0.6	0.5
Lymphocytes (%)	22.6–36.6	33.3	30.9	31.4	32.8	31.6
Monocytes (%)	4.7–8.7	7.2	6.7	6.5	6.3	6.5
Iron (serum) (µg/dL)	75–150	131	88	95	92	87
Glucose (mg/dL)	70–100	86	86	89	87	86
Cholesterol Total (mg/dL)	<190	167	164	164	165	164
HDL-c (mg/dL)	>40	60	57	62	59	60
LDL-c (mg/dL)	<129	94	97	92	93	96
VLDL-c (mg/dL)	<15	13	10	10	11	10
Triglycerides (mg/dL)	<150	63	52	51	51	52
Potassium (mEq/L)	3.5–5.1	5.5	4.7	5.9	5	4.9
Sodium (mEq/L)	135–147	142	142	141	142	141
Chlorides (mEq/L)	98–106	102	102	101	102	101
CK (U/L)	35–232	291	602	602	579	604
AST (U/L)	8–48	33	41	39	40	41
ALT (U/L)	7–55	32	32	33	33	34
Gamma GT (U/L)	8–61	16	18	16	18	16
C-Reactive Protein (mg/dL)	<1.0	0.01	0.01	0.01	0.01	0.01
Cortisol (nmol/L)	140–690	522	525	576	578	575
Testosterone (nmol/L)	8.7–34.7	29.4	31.2	30.8	30.4	29.8
Testosterone/Cortisol Ratio		0.056	0.059	0.053	0.052	0.051
Epinephrine (ng/L)	0–140	13	-	-	-	37
Norepinephrine (ng/L)	70–1700	283	-	-	-	287
Dopamine (ng/L)	0–30	2	-	-	-	2

Abbreviations: ALT: alanine aminotransferase; AST: aspartate aminotransferase; C: cortisol; CK: creatine kinase; HDL-c: High-density lipoprotein cholesterol; LDL-c: low-density lipoprotein cholesterol; MCH: mean corpuscular hemoglobin; MCHC: mean corpuscular hemoglobin concentration; MCV: mean corpuscular volume; T: testosterone; T/C ratio: testosterone/cortisol ratio; VLDL-c: very-low-density lipoprotein cholesterol.

## Data Availability

The data presented in this study are only available upon request from the corresponding author. The data are not publicly available as they contain information that could compromise the privacy of study’s participant.

## Abbreviations

The following abbreviations are used in this manuscript:

ALT	Alanine aminotransferase
AST	Aspartate aminotransferase
C	Cortisol
CK	Creatine Kinase
DRI	Dietary Reference Intake
HDL-c	High-density lipoprotein cholesterol
HPLC	High-performance liquid chromatography
LDL-c	Low-density lipoprotein cholesterol
MCH	Mean corpuscular hemoglobin
MCHC	Mean corpuscular hemoglobin concentration
MCV	Mean corpuscular volume
SPM	Strokes per minute
T	Testosterone
T/C ratio	Testosterone/cortisol ratio
VLDL-c	Very-low-density lipoprotein cholesterol
